# Editorial: Transient receptor potential channels (TRP): signal transduction

**DOI:** 10.3389/fmolb.2023.1201614

**Published:** 2023-04-28

**Authors:** Alexander Dietrich, Erik J. Behringer, Mark S. Taylor, Swapnil K. Sonkusare

**Affiliations:** ^1^ Walther-Straub-Institute of Pharmacology and Toxicology, Member of the German Centre for Lung Research (DZL), Munich, Germany; ^2^ Loma Linda University, Loma Linda, CA, United States; ^3^ University of South Alabama, Mobile, AL, United States; ^4^ University of Virgina, Charlottesville, VA, United States

**Keywords:** transient receptor potential channels, TRPA1, TRPV1, extracellular matrix (ECM), osteoarthritis (OA), endothelial cells, hypertension-induced hemorrhagic and ischemic stroke

The mammalian superfamily of transient receptor potential (TRP) channels covers 28 members grouped in six families: TRPC, with C for “canonical”; TRPA, with A for “ankyrin”; TRPM, with M for “melastatin”; TRPML, with ML for “mucolipidin”; TRPP, with P for “polycystin”; and TRPV, with V for “vanilloid”. They all share a structure of six transmembrane (TM) regions, with a pore domain between TM5 and 6 and cytoplasmic amino- and carboxyl-termini. Functionally non-selective, Ca^2+^ permeant TRP channels are tetramers, which consist of the same or different TRP monomers, preferentially from the same family. While equipped with a ≥10,000-fold transmembrane concentration gradient, Ca^2+^ influx through open Ca^2+^-permeant TRP channels serves a dual role by (i) sustaining the activation of intracellular enzymes and plasma membrane ion channels and (ii) continuously replenishing the endoplasmic reticulum Ca^2+^ supply for consistent and repetitive physiological functions. This latest Research Topic of Frontiers in Molecular Biosciences on TRP signal transduction has centered its discussion on two Ca^2+^-permeant TRP channels, namely, TRPA1, and TRPV1.

TRPA1 is the only member of the TRPA family and contains a characteristic high number of N-terminal ankyrin repeats (16 in humans), from which it derives its name. Ankyrin repeats are 33-amino acid motifs, which are found in many proteins where they mediate protein–protein interactions. The channel was originally cloned in human fibroblasts, where it inhibits fibroblast-to-myofibroblast differentiation, a hallmark of lung fibrosis. Myofibroblasts produce the extracellular matrix (ECM), which is important for cell barrier function but inhibits gas exchange in patients suffering from pulmonary fibrosis. Next to these cells, chondrocytes also produce ECM to keep the cartilage environment stable, which is, however, seriously damaged during osteoarthritis (OA), a chronic inflammatory destruction of joints. The authors of a manuscript of this Research Topic tested the hypothesis of whether activation of TRPA1 by one of its agonists, allyl isothiocyanate (AITC), changes the secretion of ECM and inflammation-related interleukins (Che et al.). Indeed, TRPA1 protein and mRNA were upregulated in the cartilages of patients with OA, and their chondrocytes developed a different swollen shape corresponding to a fibrotic phenotype. In an *in vitro* model, they cultured chondrocytes from OA patients on stiff (plastic) and soft (hydrogel) substrates and showed a significantly higher intracellular Ca^2+^ concentration ([Ca^2+^]_i_) during the culture time and after application of AITC. Both TRPA1 expression and [Ca^2+^]_i_ were, however, not different when comparing the two different substrates. After application of AITC, both positive and harmful effects were observed on a softer and stiffer matrix, respectively. Production of collagen I, as an important ECM protein, and the nuclear localization of its transcription factor MRTF-A (myocardin-related transcription factor A) were elevated after application of AITC in chondrocytes grown on a stiff but not on a soft matrix, while the secretion of both an inflammatory and anti-inflammatory marker at IL-6 and IL-10 levels, respectively, was higher on a softer substrate (Che et al.). Although additional evidence, e.g., by siRNA-mediated downregulation of the TRPA1 protein, is unfortunately missing, one can speculate that TRPA1 activation and a softer environment may be beneficial for patients suffering from osteoarthritis.

Next to AITC, TRPA1 channels are also activated by a plethora of different compounds including lipid peroxide metabolites generated by reactive oxygen species (ROS), which are produced during uncontrolled hypertension. In a second manuscript, the role of TRPA1 on hypertension-induced hemorrhagic stroke was analyzed (Sullivan et al.). Hypertension was induced by a combination of the application of angiotensin II, a nitric oxide synthase inhibitor, and a high-salt diet in mice with a tissue-specific knockout of TRPA1 channels in endothelial cells (*Trpa1-*ecKO mice) and floxed control mice (*Trpa1* ^fl/fl^). Both *Trpa1*-ecKO and *Trpa1* ^fl/fl^ mice became hypertensive, and a similar majority developed intracerebral hemorrhage lesions, which were significantly smaller in *TrpA1*-ecKO. However, morbidity and mortality were not different between the groups (Sullivan et al.). After the identification of a neuroprotective effect during ischemic stroke induced by the hypoxia-induced dilation of cerebral arteries by TRPA1 channels, the authors demonstrated no significant similar impact of TRPA1 on a hypertension-induced hemorrhagic stroke. Therefore, these data demonstrate well a very specific role of TRPA1 channels in the protection against ischemic but not hemorrhagic stroke.

TRPV1 was the first of six TRPV subtypes to be discovered in 1997 when extracted from rodent dorsal root ganglia (DRG) and cloned in HEK293 cells. Tetrameric ion channels comprising only TRPV1 isoforms have a high permeability to Ca^2+^ (P_Ca_/P_Na_≈10). TRPV1-containing channels are classically activated by capsaicin enriched in hot chili peppers. Consistently with their original identification in DRG neurons, TRPV1 channels have been primarily associated with nociception. Using potent capsaicin mimetics (resiniferatoxin, n-oleoyl-dopamine), Maggi et al. identified a novel role for TRPV1 as a doorway for the apoptosis of chronic myeloid leukemia cells via Ca^2+^ influx and oxidative stress. Further, they found that this effect was synergistic with the co-application of the FDA-approved drug imatinib.

In summary, TRP channels are involved in many cellular signaling pathways and are integral to physiology of blood flow control, neurotransmission, the deveolpment of inflammation and diseases such as cancer. Future research will further reveal them as important pharmacological targets for new and more efficient therapeutic options.

